# Supplementary material for: Advances in AI for Protein Structure Prediction: Implications for Cancer Drug Discovery and Development
Source: Biomolecules. 2024 Mar 12;14(3):339. doi: 10.3390/biom14030339 (PMC10968151; doi:10.3390/biom14030339)
Supplement: Supplementary file 1 [file biomolecules-14-00339-s001.zip › biomolecules-2854562-supplementary.pdf]

Scheme 1. Comparison of algorithms among AF2, ESM2 and OpenFold.

	**AlphaFold2**	**ESM2**	**OpenFold**
Model Structure	b+c+d	a+e+f	b+c+d
Parameters	21 million	15 billion	93 million
Framework implementation	TensorFlow, Jax	PyTorch	PyTorch
Uses MSA	☑		☑
Uses Structure templates	☑		☑
Uses sequence	☑	☑	☑

a. Sequence input.

b. Three channel input.

c. Transformer based prediction module.

d. 3D structure equivalent module.

e. Transformer based prediction model without using MSA.

f. 3D equivalent structure module without using MSA.
